# Application of Artificial Intelligence in Vulnerable Carotid Atherosclerotic Plaque Assessment—A Scoping Review

**DOI:** 10.3390/medicina61122082

**Published:** 2025-11-22

**Authors:** Alexandros Barbatis, Konstantinos Dakis, Petroula Nana, George Kouvelos, Miltiadis Matsagkas, Athanasios Giannoukas, Konstantinos Spanos

**Affiliations:** 1 Vascular Surgery Department, University Hospital of Larissa, 41334 Larissa, Greeceagiannoukas@hotmail.com (A.G.);; 2 German Aortic Centre, Department of Vascular Medicine, University Medical Center Eppendorf, 20251 Hamburg, Germany

**Keywords:** stroke, TIA, carotid stenosis, artificial intelligence, deep learning

## Abstract

*Background and Objectives:* Accurate evaluation of vulnerable carotid atherosclerotic plaques remains essential for preventing ischemic stroke. Conventional imaging modalities such as ultrasound and computed tomography angiography (CTA) have limited capacity to identify histopathological features of plaque instability, including fibrous cap rupture, lipid-rich necrotic core, and intraplaque hemorrhage. Artificial intelligence (AI) techniques—particularly deep learning (DL) and radiomics—have recently emerged as valuable adjuncts to standard imaging, achieving AUC values of 0.83–0.99 across modalities in identifying vulnerable plaques. This scoping review summarizes the available evidence on the application of AI in the detection and assessment of vulnerable carotid plaques. *Methods:* A systematic search of the English-language literature was conducted in MEDLINE, SCOPUS, and CENTRAL from 2000 to 30 June 2025, following the PRISMA-ScR framework. Eligible studies applied AI-based approaches (machine learning, deep learning, or radiomics) to evaluate carotid plaque vulnerability using ultrasound, CTA, or MRI. Extracted outcomes included diagnostic performance, correlation with histopathology or neurological events, and predictive modeling for stroke risk. *Results:* Of 201 records screened, 12 studies met inclusion criteria (ultrasound = 6; CTA = 4; high-resolution MRI = 2; publication years 2021–2025). All reported receiver operating characteristic area-under-the-curve (ROC-AUC) values for endpoints related to plaque vulnerability (symptomatic versus asymptomatic status, presence of intraplaque hemorrhage or lipid-rich necrotic core, fibrous-cap surrogates, and, less frequently, short-term cerebrovascular events). For ultrasound, contrast-enhanced videomics achieved an AUC of 0.87 (10 centers; n = 205), B-mode texture/radiomics reached 0.87 (n = 150), and segmentation-assisted models 0.827 (n = 202); other ultrasound models reported AUCs of 0.88–0.91. For CTA, a symptomatic-plaque machine-learning model yielded AUC 0.89 ( n = 106); a perivascular-adipose-tissue (PVAT) radiomics nomogram achieved AUC 0.836 on external validation; a histology-referenced pilot attained AUC 0.987; and one mild-stenosis TIA model reported ROC performance. For high-resolution MRI (HR-MRI), radiomics-based models showed AUC 0.835–0.864 in single-modality cohorts and up to 0.984 with multi-contrast inputs. Across modalities, AUC ranges were: ultrasound 0.827–0.91, CTA 0.836–0.987 (external 0.836), and HR-MRI 0.835–0.984. Only two out of twelve studies performed external validation; calibration and decision-curve analyses were rarely provided, and most cohorts were single-center, limiting generalizability. *Conclusions:* AI demonstrates strong potential as a complementary tool for evaluating carotid plaque vulnerability, with high diagnostic performance across imaging modalities. Reported AUCs ranged from 0.83 to 0.99 based primarily on internal or hold-out validation, representing the upper bound of theoretical rather than real-world performance. Nonetheless, large prospective multicenter studies with standardized protocols, histopathological correlation, and external validation are required before clinical integration into stroke prevention pathways.

## 1. Introduction

Carotid atherosclerotic disease represents a major cause of ischemic stroke, with risk determined not only by the degree of luminal stenosis but also by the biological composition of the plaque itself. Features of plaque vulnerability—such as intraplaque hemorrhage, lipid-rich necrotic core, thin or ruptured fibrous cap, surface ulceration, and intraplaque neovascularization—are consistently associated with artery-to-artery embolism and recurrent cerebrovascular events [1,2,3].

Modern management therefore combines luminal measurements with validated imaging biomarkers to refine patient selection for either revascularization or intensified medical therapy.

Ultrasound, including contrast-enhanced ultrasound (CEUS), remains the first-line modality for quantifying stenosis, assessing echogenicity (e.g., gray-scale median), evaluating surface morphology, and visualizing plaque microvascularity [4,5]. Computed tomography angiography (CTA) provides high-resolution visualization of ulceration, surface irregularity, and calcification burden, while high-resolution magnetic resonance imaging (MRI) serves as the reference standard for component-level tissue characterization, particularly for detecting intraplaque hemorrhage [5,6,7,8,9,10]. Despite these strengths, differences in acquisition parameters, segmentation protocols, and observer interpretation continue to limit inter-center reproducibility and complicate individualized risk stratification [6,7,8].

Recent clinical guidelines from the European Society for Vascular Surgery (ESVS, 2023) and the Society for Vascular Surgery (SVS, 2022) emphasize that stroke risk depends on both stenosis severity and plaque morphology [1,2]. While these guidelines acknowledge the prognostic value of imaging features—such as echolucent plaques, ulceration, or intraplaque hemorrhage—they currently provide no framework for quantitative or AI-based assessment. This highlights a translational gap between advanced image analytics and everyday vascular practice [11].

Artificial intelligence (AI)—encompassing machine learning (ML), deep learning (DL), and radiomics—introduces a framework for standardized, quantitative analysis of routine vascular images and for integrating multimodal data to enable personalized risk prediction. In ultrasound and CEUS, AI models have been trained for automatic lumen–plaque segmentation, echogenicity quantification, and grading of neovascularization [8,11,12,13,14]. In CTA, DL and radiomics approaches have been applied to detect ulceration, quantify texture-defined vulnerability, and differentiate symptomatic from asymptomatic plaques; selected studies have explored short-term TIA or stroke prediction [15,16,17]. MRI studies were included when directly relevant to plaque vulnerability or when used as a reference standard, while purely technical sequence-optimization papers were excluded. MRI-based radiomics further allows multiparametric characterization of intraplaque hemorrhage and lipid-rich necrotic core [18,19].

Parallel advances across cardiovascular imaging strengthen the rationale for AI adoption in carotid disease. In coronary CT angiography, for example, radiomics-derived plaque signatures have improved discrimination of high-risk lesions beyond conventional metrics [20]. Similarly, AI models in abdominal aortic aneurysm surveillance have shown potential for predicting sac growth and endoleak formation after EVAR [21,22]. These successes demonstrate that data-driven image analysis can reveal subtle tissue patterns inaccessible to the human eye and may eventually complement clinical decision-making.

Nevertheless, evidence in carotid imaging remains fragmented across modalities, algorithms, and endpoints. Reported performance varies according to dataset size, imaging protocol, and validation strategy; most studies are single-center with limited external testing. Calibration, interpretability, and decision-curve analyses are inconsistently reported, restricting assessment of real-world utility [11,23]. Moreover, methodological heterogeneity—differences in feature extraction, class balance, and outcome definition—complicates comparison between models and slows regulatory acceptance.

This scoping review synthesizes published clinical studies evaluating AI in the assessment of vulnerable carotid atherosclerotic plaque, focusing primarily on ultrasound (including CEUS) and CTA, and incorporating MRI where directly relevant. Its objectives are to (1) summarize diagnostic accuracy for vulnerability features, (2) characterize predictive performance for ipsilateral TIA, stroke, or symptomatic status, and (3) describe reference standards, validation approaches, and reporting practices that determine readiness for clinical translation into guideline-directed stroke prevention.

## 2. Materials and Methods

### 2.1. Eligibility Criteria

The PRISMA Extension for Scoping Reviews (PRISMA-ScR) framework was applied [24,25] (Supplementary Table S1). This study, entitled “Application of Artificial Intelligence in Vulnerable Carotid Atherosclerotic Plaque Assessment: A Scoping Review”, was registered retrospectively at the Open Science Framework (OSF) on 14 October 2025 (https://doi.org/10.17605/OSF.IO/FBZXG, (accessed on 22 October 2025)) after searching the literature but prior to data extraction and analysis. English-language studies were eligible if they reported original clinical data on the use of artificial intelligence (AI)—including machine learning, deep learning, or radiomics—for the assessment of vulnerable carotid atherosclerotic plaque using ultrasound (including contrast-enhanced ultrasound, CEUS) or computed tomography angiography (CTA). Magnetic resonance imaging (MRI) was included when directly relevant to the evaluation of plaque vulnerability or when used as a reference standard.

Eligible outcomes comprised diagnostic performance for detecting or characterizing vulnerability features such as intraplaque hemorrhage, lipid-rich necrotic core, thin or ruptured fibrous cap, surface ulceration, intraplaque neovascularization (CEUS-IPN), or echolucency/gray-scale median, and/or predictive performance for ipsilateral transient ischemic attack (TIA), stroke, symptomatic status, or treatment-related outcomes. Systematic or narrative reviews were excluded from the quantitative synthesis but were secondarily consulted to contextualize methodological quality and reporting trends. These sources are described separately in the Results for reference only and were not included in data extraction. Exclusion criteria included reviews, editorials, letters, conference abstracts, preprints without peer review, animal or phantom experiments, non-English publications, studies focused solely on image reconstruction, denoising, or dose-reduction without plaque assessment, and studies centered on non-carotid vascular territories or unrelated indications.

### 2.2. Search Strategy

A structured literature search of MEDLINE (PubMed), Scopus, and the Cochrane Central Register of Controlled Trials (CENTRAL) was conducted for publications between 1 January 2000 and 30 June 2025. Keywords and, where applicable, Medical Subject Headings (MeSH) terms were combined using Boolean operators, including “carotid artery”, “carotid atherosclerosis”, “carotid endarterectomy”, “vulnerable carotid plaque”, “carotid stenosis”, “carotid ultrasound”, “carotid angiography”, “contrast-enhanced ultrasound”, “carotid CTA”, “computed tomography angiography”, “carotid MRI”, “magnetic resonance imaging”, “artificial intelligence”, “machine learning”, “deep learning”, and “radiomics”. Database-specific search strings and Boolean combinations are detailed in Supplementary Table S2. Duplicate records were removed using EndNote (Clarivate Analytics). Title and abstract screening, followed by full-text review, were performed independently by two investigators (A.B. and K.D.), with any discrepancies resolved by a third investigator (K.S.). The reference lists of included articles were also manually reviewed to identify additional relevant studies.

### 2.3. Data Extraction

Data were extracted into a standardized Microsoft Excel spreadsheet. For each study, bibliographic details (authors, title, journal, year), study design (single or multicenter, prospective or retrospective), and population characteristics (age, sex, vascular risk factors, symptom status) were recorded when available. Imaging protocols were documented by modality: for ultrasound/CEUS, key acquisition parameters and CEUS use; for CTA, contrast injection protocol, reconstruction settings, and any dual-energy or spectral configuration; and for MRI, field strength and specific sequences used for component-level phenotyping.

Each AI approach was characterized according to its analytic task, model type, dataset size and partitioning, internal or external validation strategy, and whether it involved multicenter or multivendor data. The reference standard was classified as histopathology, MRI-based component identification, symptomatic status, or clinical event correlation. Extracted outcomes included diagnostic metrics such as sensitivity, specificity, accuracy, and area under the receiver-operating characteristic curve (AUC). When available, data on predictive performance for ipsilateral TIA, stroke, or symptomatic status were also recorded. Indicators of model calibration and clinical utility, such as reclassification analysis or decision-curve analysis, as well as comparisons with clinician or standard-of-care interpretation, were included when reported.

### 2.4. Definitions

“Symptomatic carotid disease” was defined as recent ipsilateral TIA, amaurosis fugax, or ischemic stroke, while “asymptomatic disease” denoted the absence of these events within the time interval specified by the source study. “Carotid stenosis” was expressed as the percentage of luminal narrowing using NASCET-equivalent methodology when reported. “Vulnerable plaque features” included intraplaque hemorrhage, lipid-rich necrotic core, thin or ruptured fibrous cap, surface ulceration, intraplaque neovascularization, and echolucency/gray-scale median, according to the criteria of each study.

AI tasks were categorized as segmentation (lumen, plaque, or component delineation), characterization (feature detection or classification, including vulnerability labeling), or prediction (ipsilateral TIA/stroke, symptomatic status, or treatment-related outcomes). Validation was considered internal when model performance was estimated through resampling or hold-out testing within a single dataset and external when assessed using an independent cohort differing by time, site, or vendor. Diagnostic performance referred to sensitivity, specificity, accuracy, and AUC as reported by each study.

### 2.5. Data Analysis

Given the expected heterogeneity in study design, patient populations, imaging protocols, labeling strategies, and validation methodologies, no quantitative meta-analysis was performed. Across modalities, pooled synthesis was not feasible owing to heterogeneity in endpoints and AI methodologies; nevertheless, observed AUCs consistently exceeded 0.80, indicating strong diagnostic performance of AI-enhanced imaging. When numerical data were available for comparable AI tasks and reference standards, results were summarized descriptively in tabular format. Otherwise, findings were synthesized narratively, with structured mapping of study characteristics, AI methodologies, and reported outcomes.

### 2.6. Ethics

This review analyzed the previously published literature and did not require institutional ethics approval. It was conducted in accordance with the principles of the Declaration of Helsinki.

## 3. Results

### 3.1. Study Selection

**Figure 1:**
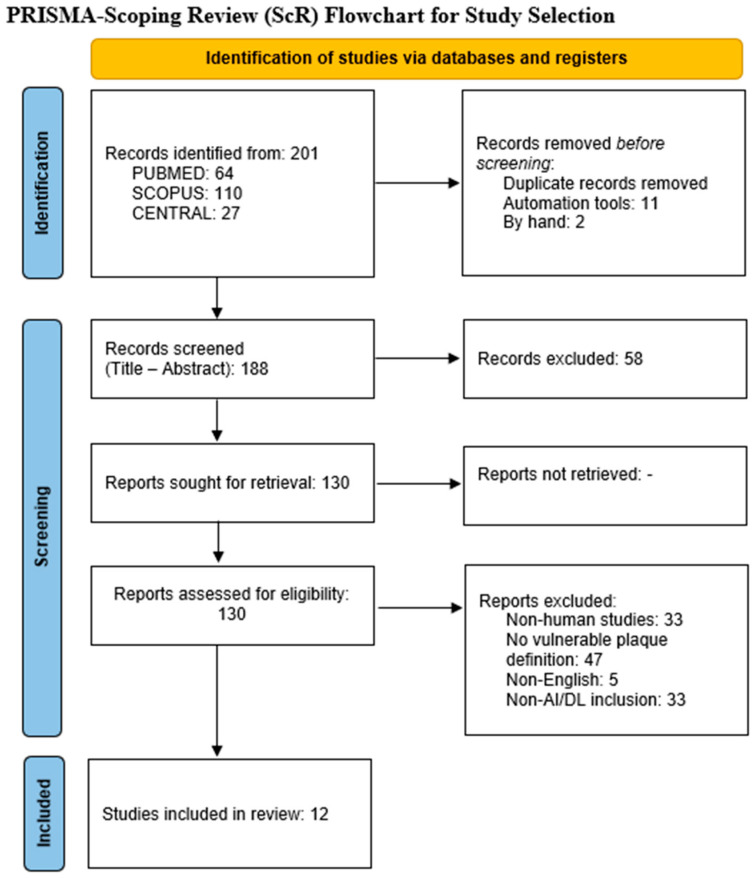
PRISMA 2020 flowchart.

The literature search identified 201 records. Following title and abstract screening, 12 primary studies were eligible for full-text review and qualitative synthesis, while six secondary analyses (systematic or narrative reviews) were retained for contextual and methodological appraisal, yielding a total of 18 studies included in the review (Figure 1, PRISMA 2020 flowchart). The 12 original investigations comprised AI or radiomics analyses based on ultrasound, computed tomography angiography (CTA), or high-resolution magnetic resonance imaging (HR-MRI), whereas the six secondary studies consisted of structured systematic reviews or narrative summaries addressing AI across carotid imaging modalities.

The primary dataset encompassed multicenter prospective CEUS videomics studies, single-center CTA radiomics analyses, HR-MRI radiomics investigations, and ultrasound texture-based machine-learning studies [15,16,17,18,19,26,27,28,29,30,31,32] (Table 1). The secondary set included both modality-specific and cross-modality reviews—such as CEUS deep-learning diagnostic studies contextualized by narrative appraisals, ultrasound-focused radiomics quality (RQS) meta-analyses, and broader domain reviews on AI in symptomatic or vulnerable carotid plaque [7,8,33,34,35,36,37,38].

### 3.2. Study Population and Modality Breakdown

Among the 12 primary studies, imaging modalities included CTA in four studies [15,16,17,32], HR-MRI in two studies [18,19], and ultrasound (B-mode and/or CEUS) in six studies [26,27,28,29,30,31]. Cohorts ranged from a large prospective multicenter CEUS study that screened 547 potentially eligible patients across ten hospitals (with 205 CEUS videos analyzed) to smaller single-center retrospective datasets and multicenter CTA analyses. The main endpoints involved symptomatic versus asymptomatic plaque classification, component-level vulnerability surrogates (intraplaque hemorrhage [IPH], lipid-rich necrotic core [LRNC], or fibrous-cap status), and short-term cerebrovascular events [15,16,17,18,19,26,27,28,29,30,31,32] (Table 2).

### 3.3. Reference Standards and Endpoints

Across CTA, HR-MRI, and ultrasound investigations, symptomatic status adjudicated by neurologists and expert imaging consensus frequently served as the ground truth. Histopathology provided tissue-level labels in one CTA radiomics pilot, while MRI-derived component maps (IPH/LRNC) functioned both as targets and as reference standards for sonographic texture-learning models [15,16,17,19,29].

### 3.4. Modality-Specific Outcomes

#### 3.4.1. Computed Tomography Angiography (CTA)

Machine-learning integration of degree of stenosis with 13 plaque-composition subcomponents identified plaques associated with recent cerebrovascular events with an AUC of 0.89 on an independent test cohort (n = 106), outperforming stenosis alone (AUC 0.51), IPH presence (AUC 0.69), and a conventional composition model (AUC 0.78; all *p* < 0.001) [15]. A radiomics analysis with surgical correlation (n = 30 plaques) showed strong tissue-level discrimination, where entropy and volume features achieved AUC 0.92–0.96 individually, a CART model combining both reached AUC 0.987 with 86.7% correctly classified plaques, and an SVM classifier yielded 86.7% accuracy, 92.9% precision, and 81.3% recall under five-fold cross-validation [32]. A multicenter CTA radiomics investigation of perivascular adipose tissue (PVAT) reported that a combined radiomics and clinical model achieved AUC 0.942 (training), 0.797 (internal/external validation), and 0.836 (external validation). The best individual classifier (XGBoost) reached a mean AUC of 0.797 across validation cohorts [16]. Radiologic–pathologic correlation confirmed that CTA-derived texture signatures can reproduce histology-concordant high-risk patterns, supporting the biological validity of CT-based learning features [17].

#### 3.4.2. High-Resolution MRI

Three-dimensional HR-MRI radiomics for vulnerable-plaque identification demonstrated AUC 0.915 (training) and 0.835 (testing) for radiomics-only models and 0.957/0.864 for combined clinical + radiomics models, both outperforming conventional imaging features (DeLong *p* < 0.05) [18]. A subsequent multi-contrast HR-MRI study in 162 patients reported AUC 0.988 (training) and 0.984 (test) for the radiomics model and 0.989/0.986 for the combined model, compared with 0.825/0.804 for traditional features [19]. These findings underscore the robustness of MRI-based texture and intensity signatures for high-risk plaque identification.

#### 3.4.3. Ultrasound and CEUS

A prospective multicenter CEUS videomics study across ten hospitals achieved AUC 0.85 (training) and 0.87 (hold-out), with sensitivity 79.2% and specificity 84.4% in the hold-out cohort, outperforming experienced readers and a strong static-CNN comparator [28]. A B-mode texture/radiomics model trained against HR-MRI-defined vulnerability in 150 patients yielded AUC 0.88 (training) and 0.87 (testing), C-index 0.84, and test-set accuracy 84.8%, exceeding conventional grayscale metrics [29]. An AI-assisted ultrasound segmentation + radiomics workflow in 202 ischemic-stroke patients produced AUC 0.894 (95% CI 0.807–0.981; sensitivity 79.8%; specificity 93.1%) in training and 0.827 (95% CI 0.716–0.938; sensitivity 81.8%; specificity 80.0%) in validation, confirming reproducible plaque-stability stratification through automated delineation [27]. Deep-learning analysis of routine B-mode ultrasound for internal carotid plaque classification achieved AUC ≈ 0.91, accuracy 0.88, sensitivity 0.94, and specificity 0.71, supporting its feasibility for risk-oriented triage in vascular ultrasound laboratories [30].

#### 3.4.4. Cross-Modality Synthesis and Methodological Signals

Across CTA, HR-MRI, and ultrasound modalities, integration of imaging features with clinical covariates consistently improved discrimination compared with single-source models. Where reported, calibration and decision-curve analyses supported potential clinical utility within test cohorts. However, only two of the twelve studies performed true external validation, underscoring the limited generalizability of current AI models and highlighting the need for multicenter, multivendor testing [17] (Table 3). Contemporary reviews converge on these findings, highlighting heterogeneous pipelines, variable adherence to IBSI and TRIPOD-AI reporting standards, and a persistent need for multicenter external validation. Nevertheless, the collective evidence indicates that AI-enhanced carotid imaging improves characterization of plaque vulnerability beyond stenosis-based assessment alone [11,12,13,39,40,41,42,43]. A focused synthesis on AI-based prediction in asymptomatic carotid stenosis further emphasized the importance of event-anchored endpoints, standardized acquisition and labeling, and prospective validation as prerequisites for clinical translation [36].

## 4. Discussion

Artificial intelligence (AI) in carotid imaging is converging on a clinically coherent goal: the standardized quantification of plaque biology to complement stenosis grading and refine stroke-prevention strategies. Vulnerability phenotypes–including intraplaque hemorrhage (IPH), lipid-rich necrotic core (LRNC), thin or ruptured fibrous cap, surface ulceration, intraplaque neovascularization (IPN), and echolucency or gray-scale median (GSM)—are consistently associated with ipsilateral cerebrovascular events and are increasingly reflected in contemporary guideline discussions and structured-reporting initiatives [1,2,3,4,5,6,7].

In ultrasound and CEUS applications, AI pipelines typically combine automated plaque segmentation with quantitative descriptors of echogenicity and microbubble kinetics to estimate IPN. Across multiple studies, directionally consistent findings have emerged: lower echogenicity (GSM) and higher IPN grades correlate with symptomatic status and other indicators of plaque instability, consistent with established pathophysiology and meta-analytic evidence. Given that ultrasound is portable, repeatable, and cost-effective, these AI-derived targets are clinically attractive. Nevertheless, rigorous implementation depends on controlled acquisition parameters and normalized input (for example, fixed dynamic-range or gain settings, mechanical-index reporting for CEUS) and on standardized feature extraction and processing when radiomics is applied [1,4,5,10].

In CTA, AI efforts focus on features with direct procedural and prognostic relevance, including ulceration and surface complexity, low-attenuation zones as surrogates for LRNC, calcification morphology, and the emerging role of perivascular adipose tissue (PVAT) radiomics as a potential marker of perilesional inflammation. Radiomics and interpretable machine-learning models have consistently improved symptomatic versus asymptomatic classification compared with visual assessment, while early deep-learning systems demonstrate automated plaque detection and triage on routine angiograms. Spectral or dual-energy acquisitions further expand the quantitative domain and may enhance discrimination between lipid and fibrous components. Concordance strengthens when CTA labels are anchored to MRI-defined IPH or CEUS-defined IPN, linking model outputs to biological substrates already associated with clinical events [3,6,7,8,10]. In practice, CTA’s speed, accessibility, and high-resolution depiction of ulceration position it as a natural complement to ultrasound in acute evaluation and pre-procedural planning [1,6,7].

Across both modalities, predictive performance improves when imaging features are combined with clinical variables rather than analyzed in isolation. Models integrating AI-derived plaque metrics with age, vascular risk factors, and symptom timing outperform stenosis-only baselines for predicting ipsilateral TIA, stroke, or symptomatic status. These results support clinically tangible use cases aligned with existing guideline frameworks: prioritizing recently symptomatic patients for expedited revascularization when vulnerability features are present; reclassifying asymptomatic patients into higher near-term risk strata when high-risk plaque biology is detected despite comparable stenosis; and informing procedural choice (CEA versus CAS) through standardized quantification of ulceration and calcification distribution [1,2,6,7].

Recent systematic reviews and meta-analyses reinforce these observations. Hou et al. summarized CEUS and radiomics studies evaluating intraplaque neovascularization, reporting pooled AUCs between 0.84 and 0.90 for discriminating vulnerable from stable plaques [11]. Vacca et al. conducted a quantitative synthesis of ultrasound-based radiomics, yielding comparable pooled AUCs (0.82–0.91) and emphasizing the limited external validation across studies [34]. In CTA, pooled estimates from Biswas et al. and Cilla et al. demonstrated average AUCs of 0.86–0.93 for radiomics models identifying high-risk plaques, consistent with the modality ranges observed in the present review (CTA 0.836–0.987; US 0.827–0.91; HR-MRI 0.835–0.984) [32,38]. These analyses also highlighted recurrent methodological challenges—small datasets, absence of external testing, and variable Radiomics Quality Scores (RQS < 15 in most studies). By incorporating studies published through mid-2025, the present scoping review extends this evidence base, capturing videomic CEUS pipelines and PVAT-radiomics models not previously represented. Collectively, these convergent findings confirm that AI-enhanced carotid imaging provides reproducible, modality-independent signals of plaque vulnerability, while underscoring the need for harmonized protocols and transparent validation to enable clinical translation.

Because most models were internally validated, reported AUCs should be interpreted as upper-bound estimates; true clinical performance will depend on independent external validation. Only a minority of studies reported calibration, interpretability, or decision-curve analyses (DCA). These elements are essential to demonstrate clinical utility beyond accuracy metrics; future research should systematically include these assessments. Decision-curve analysis quantifies the net clinical benefit of a model across threshold probabilities, directly linking diagnostic output to patient-level outcomes. Unlike AUC, DCA captures benefit–harm trade-offs and is therefore indispensable for evaluating real-world clinical utility.

For translation into clinical practice, methodological discipline remains essential. Studies defining explicit reference standards—histopathology where feasible, or alternatively MRI-derived IPH or adjudicated symptomatic status—together with robust pre-processing, leakage control, and validation beyond a single split, provide the most credible evidence. Progressing from internal (TRIPOD Level 2) to external or prospective (Levels 3–4) validation requires standardized imaging protocols, scanner/vendor harmonization, independent temporal or geographic cohorts, and full reporting of calibration, decision-curve, and reclassification analyses, as recommended by TRIPOD-AI and PROBAST-AI. Routine reporting of calibration, decision-curve or net-benefit analyses, and reclassification statistics is needed to demonstrate clinical utility rather than accuracy alone. Compliance with emerging frameworks such as CLAIM and TRIPOD-AI/PROBAST-AI for reporting and CONSORT-AI or SPIRIT-AI for interventional evaluation clarifies methodological assumptions, facilitates comparability, and accelerates regulatory and clinical adoption. Radiomics studies should also conform to IBSI standards to ensure feature harmonization and reproducibility [39,41,42,43]. Although fewer MRI-based studies were available, radiomics-derived texture features from HR-MRI achieved AUCs up to 0.984, underscoring MRI’s complementary role in multiparametric AI assessment.

Taken together, current evidence suggests that AI can standardize the assessment of vulnerable carotid plaque on ultrasound/CEUS and CTA and provide incremental improvements in risk stratification beyond stenosis alone. These findings collectively emphasize that AI augments, rather than replaces, existing imaging evaluation, offering a reproducible framework for plaque characterization within contemporary clinical pathways [1,2,8,9,10,11].

### Limitations

Most included studies were retrospective, single-center analyses with modest sample sizes, limiting causal inference and increasing susceptibility to selection bias. Considerable heterogeneity was present in the definitions and reference standards used for vulnerable-plaque features—for example, variation in MRI sequences and thresholds for intraplaque hemorrhage, differing CEUS grading scales for intraplaque neovascularization, and inconsistent CTA criteria for ulceration—introducing between-study variability and precluding quantitative pooling.

Acquisition and reconstruction protocols also differed across scanners and vendors (e.g., gain and dynamic-range settings in ultrasound/CEUS, or kernel, slice thickness, and contrast timing in CTA), raising the risk of domain shift and constraining generalizability. Reporting of AI methodology was inconsistent: several studies lacked transparent descriptions of pre-processing, feature selection, and data-leakage prevention; external validation, calibration, and decision-curve or net-benefit analyses were infrequently reported; and model or code availability was uncommon, limiting reproducibility.

Many prediction studies relied on symptomatic status as a surrogate endpoint rather than prospectively documented ipsilateral TIA or stroke, and event-based follow-up was short or absent. Restriction to ultrasound/CEUS and CTA modalities and to English-language publications may have under-represented MRI-based or non-English evidence. Finally, although the review protocol was defined a priori, it was not prospectively registered, and the scoping-review design precludes estimation of pooled effect sizes. These limitations collectively reinforce the reviewers’ concern that current AUC values largely reflect internal validation; future research must focus on multicenter, prospective datasets with standardized acquisition and TRIPOD-AI-compliant validation.

## 5. Conclusions

Artificial intelligence applied to ultrasound—including contrast-enhanced ultrasound (CEUS)—and computed tomography angiography (CTA), demonstrates robust potential to standardize the assessment of carotid plaque vulnerability and to improve risk stratification beyond conventional stenosis grading. Current evidence indicates that AI-based models enhance discrimination of component-level features and symptomatic status, supporting their role as adjunctive decision-support tools alongside established imaging and clinical evaluation. However, the apparent high diagnostic performance must be interpreted cautiously, as most studies rely on internal or hold-out validation. True clinical utility can be confirmed only through external, multicenter validation incorporating calibration and decision-curve analyses. For translation into clinical practice, future studies must emphasize prospective multicenter validation, harmonized acquisition and labeling protocols, and transparent reporting of calibration, decision-curve analyses, and net clinical benefit. Once these prerequisites are met, AI-derived plaque biomarkers could be incorporated into guideline-directed stroke-prevention pathways to refine patient selection for revascularization and optimize personalized medical therapy.

## Figures and Tables

**Table 1:** Study characteristics.

Author	Journal	Publication Year	Study Period	Type of Analysis	External Validation	Number of Participating Centers
**Zhang, X. et al. [18]**	*Front. Neurol.*	2023	2021−2022	Retrospective	No	1
**Zhang, H. et al. [26]**	*Rev. Cardiovasc. Med.*	2024	2020−2022	Retrospective	No	4
**Pisu, F. et al. [15]**	*Circ. Cardiovasc. Imaging*	2024	2019−2022	Retrospective	No	1
**Song, J. et al. [27]**	*BMC Med. Imaging*	2025	2018−2023	Retrospective	No	3
**Guang et al. [28]**	*BMJ Open*	2021	2017−2018	Prospective	No	10
**Zhang, L. et al. [29]**	*Front. Neurosci.*	2022	2016−2020	Retrospective	No	1
**Buckler, A.J. et al. [16]**	*Atherosclerosis*	2023	NR	Prospective	Yes	2
**Zhao, T. et al. [17]**	*BMC Med. Imaging*	2025	2018−2023	Retrospective	Yes	3
**Saba, L. et al. [30]**	*Int. J. Cardiovasc. Imaging*	2021	NR	Retrospective	No	1
**Zhang, R. et al. [19]**	*Eur. Radiol.*	2021	2015−2019	Retrospective	No	1
**Deng, C. et al. [31]**	*Technol. Health Care*	2023	NR	Retrospective	No	1
**Cilla, S. et al. [32]**	*Radiol. Med.*	2022	2015−2019	Retrospective	No	1

NR—not reported.

**Table 2:** Cohort demographics, imaging modality, AI type, and clinical status.

	Number of Patients	Sex Number of Males (%)	Age	Symptomatic Patients (%)	Imaging Modality	AI Type
**Zhang, X. et al. [18]**	90	58 (64.4)	58.85 ± 10.89	48 (53.3)	HR-MRI	radiomics
**Zhang, H. et al. [26]**	3683	NR	NR	0 (0	US	CNNs
**Pisu, F. et al. [15]**	268	122 (45)	76	12 (44)	CTA	ML
**Song, J. et al. [27]**	202	99 (49)	56.6 ± 1.9	202 (100)	US	ML
**Guang et al. [28]**	205	166 (80.9)	61.6 ± 8.4	67 (32.7)	CEUS video	DL
**Zhang, L. et al. [29]**	150	120 (80)	61.95 ± 9.9	150 (100)	US	ML
**Buckler, A.J. et al. [16]**	53	30 (57)	59.7	NR	CTA	DL
**Zhao, T. et al. [17]**	645	311 (48.2)	70.2	232 (35.9)	CTA	radiomics
**Saba, L. et al. [30]**	346	246 (71)	69.9 ± 7.8	196 (56.6)	US	DL + ML
**Zhang, R. et al. [19]**	162	148 (91.4)	66.8 ± 7.4	108 (66.7)	HR-MRI	radiomics
**Deng, C. et al. [31]**	-	-	-	-	US	Dense—Unet
**Cilla, S. et al. [32]**	30	19 (63.3)	72.96	0 (0)	CTA	radiomics

NR—not reported; AI—artificial intelligence.

**Table 3:** Imaging modality results.

	AUC%	Accuracy%	Sensitivity%	Specificity%
**Zhang, X. et al. [18]**	83.5	NR	93.8	63.6
**Zhang, H. et al. [26]**	91	88	94	71
**Pisu, F. et al. [15]**	89	NR	NR	NR
**Song, J. et al. [27]**	82.73	NR	81.82	80
**Guang et al. [28]**	87	NR	79.2	84.4
**Zhang, L. et al. [29]**	87	79.05	85.94	68.29
**Buckler, A.J. et al. [16]**	95-99	NR	NR	NR
**Zhao, T. et al. [17]**	NR	NR	NR	NR
**Saba, L. et al. [30]**	91	89.7	NR	NR
**Zhang, R. et al. [19]**	98.4	90.49	81.48	100
**Deng, C. et al. [31]**	-	-	-	-
**Cilla, S. et al. [32]**	98.7	NR	NR	NR

AUC—area under the curve; NR—not reported.

## Data Availability

All extracted data are included in this article and the Supplementary Material.

## Supplementary Materials

The following supporting information can be downloaded at: https://www.mdpi.com/article/10.3390/medicina61122082/s1, Table S1: PCC model; Table S2: Search strategy per protocol.
