# Supplementary material for: Application of Artificial Intelligence in Vulnerable Carotid Atherosclerotic Plaque Assessment—A Scoping Review
Source: Medicina (Kaunas). 2025 Nov 22;61(12):2082. doi: 10.3390/medicina61122082 (PMC12734408; doi:10.3390/medicina61122082)
Supplement: Supplementary file 1 [file medicina-61-02082-s001.zip › Supplementary Table S1. PCC.pdf]

Supplementary Table S1. PCC model.

**P**	**Patient**	**Patients with carotid atherosclerotic disease evaluated for plaque vulnerability.**
**C**	**Concept**	Application of **artificial intelligence (AI)** methods (machine learning, deep learning, radiomics) for **plaque detection, characterization, and prediction of vulnerability**.
**C**	**Context**	Imaging with **ultrasound (B-mode, CEUS, elastography)**, **computed tomography angiography (CTA, including dual-energy and radiomics)**, and **magnetic resonance imaging (MRI, including vessel wall and multicontrast imaging)** in human studies.
	**What type of question are you asking?**	What is the extent and nature of published research applying AI to ultrasound, CTA, and MRI for the assessment of vulnerable carotid atherosclerotic plaques?
	**Type of study you want to find**	Original human studies (prospective or retrospective observational studies, pilot trials, and validation studies), as well as reviews summarizing AI applications in carotid plaque assessment.

Footnote: The P.C.C. (patient; concept; context) model was used to define the clinical questions and clinically relevant evidence in the literature.
