# Supplementary material for: Application of Artificial Intelligence in Vulnerable Carotid Atherosclerotic Plaque Assessment—A Scoping Review
Source: Medicina (Kaunas). 2025 Nov 22;61(12):2082. doi: 10.3390/medicina61122082 (PMC12734408; doi:10.3390/medicina61122082)
Supplement: Supplementary file 1 [file medicina-61-02082-s001.zip › Supplementary Table S2. Search Strategy.pdf]

Supplementary Table S2. Search strategy per protocol.

PUBMED<br>Hits: 64	(((((((carotid artery) OR (carotid artery[MeSH Terms])) OR ((carotid artery atherosclerosis) OR (carotid artery atherosclerosis[MeSH Terms]))) OR ((carotid artery endarterectomy) OR (carotid artery endarterectomy[MeSH Terms]))) OR ((vulnerable carotid plaque) OR (vulnerable carotid plaque[MeSH Terms]))) OR ((carotid artery stenosis) OR (carotid artery stenosis[MeSH Terms]))) AND ((((((((CAROTID ULTRASOUND) OR (CAROTID ULTRASOUND[MeSH Terms])) OR ((CAROTID ARTERY ANGIOGRAPHY) OR (CAROTID ARTERY ANGIOGRAPHY[MeSH Terms]))) OR ((CONTRAST ENHANCED US) OR (CONTRAST ENHANCED US[MeSH Terms]))) OR ((CAROTID CTA) OR (CAROTID CTA[MeSH Terms]))) OR ((CAROTID COMPUTED TOMOGRAPHY ANGIOGRAPHY) OR (CAROTID COMPUTED TOMOGRAPHY ANGIOGRAPHY[MeSH Terms]))) OR ((CAROTID MRI) OR (CAROTID MRI[MeSH Terms]))) OR ((MAGNETIC RESONANCE IMAGING) OR (MAGNETIC RESONANCE IMAGING[MeSH Terms])))) AND (((((artificial intelligence) OR (artificial intelligence[MeSH Terms])) OR ((machine learning) OR (machine learning[MeSH Terms]))) OR ((deep learning) OR (deep learning[MeSH Terms]))) OR ((radiomics) OR (radiomics[MeSH Terms])))
SCOPUS<br>Hits: 110	("carotid artery" OR "carotid artery atherosclerosis" OR "carotid artery endarterectomy" OR "vulnerable carotid plaque" OR "carotid artery stenosis") AND ("CAROTID ULTRASOUND" OR "CAROTID ARTERY ANGIOGRAPHY" OR "CONTRAST ENHANCED US" OR "CAROTID

	CTA" OR "CAROTID COMPUTED TOMOGRAPHY ANGIOGRAPHY" OR "CAROTID MRI" OR "MAGNETIC RESONANCE IMAGING") AND ("artificial intelligence" OR "machine learning" OR "deep learning" OR "radiomics")
CENTRAL<br>Hits: 27	#1 carotid artery<br>#2 carotid artery atherosclerosis<br>#3 carotid artery endarterectomy<br>#4 vulnerable carotid plaque<br>#5 carotid artery stenosis<br>#6 CAROTID ULTRASOUND<br>#7 CAROTID ARTERY ANGIOGRAPHY<br>#8 CONTRAST ENHANCED US<br>#9 CAROTID CTA<br>#10 CAROTID COMPUTED TOMOGRAPHY ANGIOGRAPHY<br>#11 CAROTID MRI<br>#12 MAGNETIC RESONANCE IMAGING<br>#13 artificial intelligence<br>#14 machine learning<br>#15 deep learning<br>#16 radiomics<br>#17 {OR #1-#5}<br>#18 {OR #6-#12}<br>#19 {OR #13-#16}<br>#20 {AND #17-#19}

Total Hits: 201

Duplicate Screening:

Automatic Tool Duplicate Removal: 11

By-Hand Duplicate Removal: 2

Title-Abstract Screening: 188 (58 excluded)

Full-text Screening: 130 (118 excluded)
